# Associations of excessive sleepiness with sleep apnea physiological endo-phenotypes: the multi-ethnic study of atherosclerosis

**DOI:** 10.1093/annalsats/aaoag078

**Published:** 2026-04-07

**Authors:** Cecilia Castro-Diehl, Raichel Alex, Ali Azarbarzin, Ying Zhang, Andrew Wellman, Tianyi Huang, Scott Sands, Susan Redline

**Affiliations:** Department of Medicine, Division of Sleep and Circadian Disorders, Brigham and Women’s Hospital, Harvard Medical School, Boston, MA, United States; Department of Neurology, Division of Sleep and Circadian Disorders, Brigham and Women’s Hospital, Harvard Medical School, Boston, MA, United States; Department of Medicine, Division of Sleep and Circadian Disorders, Brigham and Women’s Hospital, Harvard Medical School, Boston, MA, United States; Department of Neurology, Division of Sleep and Circadian Disorders, Brigham and Women’s Hospital, Harvard Medical School, Boston, MA, United States; Department of Medicine, Division of Sleep and Circadian Disorders, Brigham and Women’s Hospital, Harvard Medical School, Boston, MA, United States; Department of Neurology, Division of Sleep and Circadian Disorders, Brigham and Women’s Hospital, Harvard Medical School, Boston, MA, United States; Department of Medicine, Division of Sleep and Circadian Disorders, Brigham and Women’s Hospital, Harvard Medical School, Boston, MA, United States; Department of Neurology, Division of Sleep and Circadian Disorders, Brigham and Women’s Hospital, Harvard Medical School, Boston, MA, United States; Department of Medicine, Division of Sleep and Circadian Disorders, Brigham and Women’s Hospital, Harvard Medical School, Boston, MA, United States; Department of Neurology, Division of Sleep and Circadian Disorders, Brigham and Women’s Hospital, Harvard Medical School, Boston, MA, United States; Laboratory of Epidemiology and Population Sciences, Intramural Research Program, National Institute on Aging, Baltimore, MD, United States; Department of Medicine, Division of Sleep and Circadian Disorders, Brigham and Women’s Hospital, Harvard Medical School, Boston, MA, United States; Department of Neurology, Division of Sleep and Circadian Disorders, Brigham and Women’s Hospital, Harvard Medical School, Boston, MA, United States; Department of Medicine, Division of Sleep and Circadian Disorders, Brigham and Women’s Hospital, Harvard Medical School, Boston, MA, United States; Department of Neurology, Division of Sleep and Circadian Disorders, Brigham and Women’s Hospital, Harvard Medical School, Boston, MA, United States

**Keywords:** epidemiological study, endotype traits, obstructive sleep apnea, sleepiness

## Abstract

**Background:**

Obstructive sleep apnea (OSA) is a heterogenous disease characterized by several endo-phenotypes (mechanistic traits and physiological severity metrics) and variable associations with excessive daytime sleepiness (EDS). Examining the relationship of EDS with features of OSA may identify physiological processes that drive increased risk for adverse health outcomes in patients with OSA and EDS.

**Research Question:**

Do variations in physiological endo-phenotypes identified from polysomnography associate with EDS in a general population, and do such associations vary by sex?

**Study Design and Methods:**

Participants in the Multi-ethnic Study of Atherosclerosis (MESA) Exam 5 Sleep Study underwent polysomnography, actigraphy, and questionnaire assessment. Among participants with an apnea–hypopnea index > 5 events/hour (*n* = 1783; mean, age 68.7 ± 9.1; 52% female), we examined associations between OSA endo-phenotypes including physiological severity measures (hypoxic burden, arousal intensity, event duration, heart rate response [Δ HR]), and endotypes (mechanistic traits: collapsibility, compensation, loop gain, and arousal threshold) with EDS (Epworth Sleepiness Scale, ESS > 10). Poisson regression models with robust variance were used, adjusting for demographics, smoking, and other factors.

**Results:**

EDS was found in 14% of the sample. An interquartile increase in hypoxic burden was associated with a 28% higher prevalence ratio (PR) of EDS (PR = 1.28; 95% CI: 1.10, 1.51) in models adjusted for demographic factors and smoking. An increased adjusted PR of EDS was also associated with higher loop gain (1.26; 1.12, 1.42) and higher ΔHR (1.32; 1.01, 1.73). In sex-stratified analyses, EDS was associated with shorter respiratory event duration and higher compensation in females, and with hypoxic burden and high ΔHR in males (*P* value for sex interaction < .05 for event duration). Associations did not materially change with adjustment for short sleep duration.

**Interpretation:**

EDS was associated with several OSA endo-phenotypes. EDS was more strongly associated with shorter event duration in females, with trends for stronger associations for hypoxic burden and ΔHR in males. The physiological correlates of EDS in people with OSA may contribute to differences in OSA outcomes across symptom groups.

## Introduction

Obstructive Sleep Apnea (OSA) is characterized by repeated episodes of recurrent upper airway obstruction that often results in non-restorative sleep and excessive daytime sleepiness (EDS).^1^ Among patients with OSA, EDS negatively impacts quality of life, impairs cognitive function, decreases work productivity, contributes to mood disorders, and increases risk of motor vehicle crashes.^2^ Treatment of OSA often improves EDS and related symptoms,^3^ supporting consideration of EDS as a marker to identify patients likely to benefit from OSA treatment. Symptom-based cluster analysis additionally has identified a “sleepy EDS” subtype that predicts increased incidence of cardiovascular disease.^4^ This finding suggests that EDS also may identify patients with OSA at increased risk for adverse health outcomes. Because sleepiness is a symptom rather than a direct causal mechanism of cardiovascular disease (CVD), it is critical to determine whether EDS marks specific physiological features of OSA. This information may provide insights into the physiological disturbances that patients with both OSA and EDS experience, as well as suggest specific intervention targets for improving OSA-related outcomes.

The current OSA disease defining metric—the apnea–hypopnea index (AHI)—shows only a weak correlation with EDS,^5^ underscoring that the AHI incompletely characterizes the symptom profile and heterogeneity of OSA. Notably, advances in physiological phenotyping suggest that OSA consists of multiple subtypes driven by different mechanistic traits such as pharyngeal collapsibility, reduced dilator muscle compensation, elevated loop gain (LG; i.e., greater ventilatory control sensitivity due to chemoreflex or pulmonary mechanisms), and higher arousal threshold.^6^ These subtypes likely influence various physiological responses to apnea and hypopnea events, including hypoxic burden (HB), heart rate response (ΔHR), and arousal intensity.^7^ Although previous studies have examined links between risk factors for sleepiness and the effect of PAP treatment,^8^ they often overlooked differences in this spectrum of OSA polysomnographic features that may affect outcomes and treatment response. Understanding the role of EDS as a marker of OSA heterogeneity and adverse outcomes requires elucidating its associations with OSA mechanistic traits and physiological phenotypes (together referred to as “endo-phenotypes”). As the pathophysiology of OSA and OSA-related outcomes differ between males and females, there is also a need to examine associations of EDS with physiological factors by sex.^9^

We hypothesized that differences in EDS among individuals with OSA may be attributed to variations in underlying mechanistic traits and OSA physiological severity metrics. We also examined whether these associations differ by sex and whether they persisted after considering potential confounding due to short sleep duration.

## Methods

### Study population

Data were derived from the Multi-ethnic Study of Atherosclerosis (MESA), a community-based study designed to investigate subclinical CVD among individuals aged 45 to 84 years at recruitment, who self-identified as Asian/Chinese, Black, Hispanic, or White participants. Between 2000 and 2002, MESA enrolled 6814 subjects free of known CVD. Participants returned for subsequent examinations. Institutional Review Boards in each institution approved the study, and participants signed informed consent.

At Exam 5 (2010-2013), a subgroup of MESA participants (n = 2237) was enrolled in the Sleep Study, which included overnight unattended polysomnography (PSG), 7-day actigraphy, and questionnaires that included the ESS.^10^ Participants regularly using oral airway devices, nocturnal oxygen, or continuous positive airway pressure (*n* = 95) were excluded. For the current analysis, we only included participants with an AHI of > 5 events/hour. Excluding participants with missing information on sleepiness (*n* = 24) or having a total sleep time on PSG of less than 2 h (*n* = 11) left 1783 participants for our analysis.

#### Sleep measurements

Participants underwent home polysomnography using a 15-channel type 2 monitor (Somte System; Compumedics, Abbotsville, Australia). The recording montage included central and occipital electroencephalography, bilateral electrooculograms, bipolar electrocardiogram, chin electromyography, thoracic and abdominal respiratory inductance plethysmography, bilateral limb movements, and finger pulse oximetry. Airflow was measured by thermocouple and nasal pressure cannula. Studies were scored using standardized methods as previously described.^11^ The AHI included all obstructive apneas and hypopneas with > 3% oxygen desaturation or arousal (AASM Manual for Scoring Sleep and Respiratory Events; 2012; version 2.5). Sleep endo-phenotypes were derived by applying advanced signal processing methods to these PSG data, as detailed before.^6^^,^^12-14^

Participants were asked to wear a wrist actigraph (Actiwatch Spectrum, Philips Respironics, Murrysville, PA) on their non-dominant wrist for seven consecutive days. Actigraphy data were collected and processed according to previously established protocols.^11^

#### Main exposures

1. *Sleep apnea severity measures included*:
  (a) Hypoxic Burden (HB, [%min/hr]): the area under the respiratory event-related desaturation curve, capturing the frequency, duration, and depth of desaturation with each respiratory event.^13^
  (b) Heart Rate Response to events (ΔHR beats/min): the difference between the maximum and minimum pulse rate during a subject-specific search window, extending from the pre-event minimum to the post-event minimum of each respiratory event.^15^
  (c) Arousal Intensity: a measure of change in EEG spectral power for manually detected arousals (versus a pre-arousal baseline).^12^
  (d) Event Duration (seconds): measured from the flow signal and defined as the mean duration of all apneas and hypopneas.^16^^,^^17^
2. *OSA mechanistic traits included*:
  (e) Passive Collapsibility (Vpassive, units of ventilation; %eupnea): the level of ventilation at normal or eupneic ventilatory drive.^18^ Lower values indicate greater collapsibility.^19^
  (f) Compensation (%eupnea): the difference between passive collapsibility and active collapsibility (level of ventilation when the ventilatory drive is at the arousal threshold).^18^
  (g) Loop Gain (LG; also referred to as “LG1”)—the magnitude of ventilatory drive response to a change in ventilation; higher LG indicates increased respiratory control instability.^20^
  (h) Arousal threshold (%eupnea): the level of ventilatory drive that causes arousal from sleep.^17^^,^^21^

##### Dependent variable

EDS was characterized using the ESS score dichotomized as values greater than 10.^10^^,^^22^

### Statistical analysis

The primary model was adjusted for age, sex, and self-reported race and ethnic group (White participants [reference group]), and current smoking status (a potential risk factor for endo-phenotypes and sleep fragmentation). A subsequent model additionally adjusted for short sleep duration, a potential sleep-related confounder, defined as < 6 versus ≥ 6 h, and determined by actigraphy-measured average sleep duration. If actigraphy data were unavailable, self-reported average sleep duration was used (*n* = 73). In an extended model, additional adjustments were made for co-morbid conditions, including body mass index (BMI, kg/m^2^), depressive symptoms (assessed using the Center for Epidemiological Studies Depression Scale [CES-D]),^23^ diabetes (defined as physician-diagnosed, fasting glucose levels ≥ 125 mg dL^−1^, or use of hypoglycemic medications),^24^ and prevalent CVD, adjudicated through medical record review of events occurring on or before the MESA Sleep Exam.^25^ Sex differences were assessed by conducting sex-stratified analyses and by modeling interaction terms in the overall sample.

To address skewness, several metrics underwent transformation. HB was logarithmically transformed. Vpassive (reflecting collapsibility) was constrained between 0.5% and 99.5% and transformed as previously described,^14^ and subtracted from 1 such that higher values represented greater collapsibility. The arousal threshold was constrained to 100% and then square-root transformed. Because both low and high ΔHR have been associated with CVD,^15^ ΔHR was categorized as low (<5.8 BPM, roughly the 25% percentile) and high (10.1 beats per minute, approximately the 75% percentile), with the midrange serving as the reference. Muscle compensation (Vcomp) displayed a U-shaped association with EDS. Categorizing compensation into tertiles allowed comparison of the lowest and highest tertiles against the middle.

We fitted Poisson regression models with a log link and robust (sandwich) standard errors to estimate PRs and 95% confidence intervals (CIs).^26^ Analyses were conducted using the *Hmisc* and *rms* packages in R. For continuous exposures, interquartile range (IQR) partial effects were estimated using the summary: rms function,^27^ comparing the predicted prevalence of the outcome for an increase in the exposure from the 25th to the 75th percentile (interquartile interval) while holding all other covariates constant. This approach describes the strength of association when moving from a typical low level (25th percentile) to a typical high level (75th percentile) for continuous predictors that are monotonically related to the outcome. It is useful when the minimal meaningful change in the exposure is not known a priori.^27^

### Sensitivity analyses

In sensitivity analysis, we explored whether the effect estimates for the primary models changed when considering use of psychotropic medications or depressive symptoms (defined by a binary variable that reflected use of antidepressants, anti-psychotics, and benzodiazepines or CES-D Depression Scale score ≥16), or current alcohol use.

For representative analyses, we also calculated the estimated predicted probability of EDS across the interquartile range and then computed the absolute risk difference (RD) in percentage points with 95% CI (parametric bootstrap).

## Results

Table 1 presents the sample characteristics overall and stratified by sex. Participants were an average age of 69 years and 51.7% were female. The median AHI was 21 events per hour, and 14% of the sample was classified with EDS. Compared to females, males exhibited higher AHI values, greater airway collapsibility, elevated loop gain, higher arousal threshold, longer event duration, higher HB, and a higher prevalence of increased heart rate response. Males had a higher prevalence of short sleep duration and CVD and lower depressive symptoms than females. No sex differences were observed for EDS prevalence.

**Table 1:** Characteristics of MESA sleep study participants, overall and by sex.

	Total *n* = 1783	Female *n* = 921	Male *n* = 862
**Age, yr, mean (SD)**	68.7 (9.1)	68.7 (9.0)	68.6 (9.2)
**Race/ethnicity, *n* (%)**			
** White**	655 (37)	335 (36)	320 (37)
** Chinese**	212 (12)	99 (11)	113 (13)
** Black**	483 (27)	265 (29)	218 (25)
** Hispanic**	433 (24)	222 (24)	211 (24)
**BMI, kg m^−2^ mean (SD)**	28.9 (5.5)	29.5 (6.2)	28.3 (4.5)
**Smoking status, *n* (%)**			
** Current**	130 (7)	64 (7)	66 (8)
** Noncurrent**	1641 (93)	850 (93)	791 (92)
**Depression symptoms,^a^ median (IQR)**	6 (3, 12)	6 (3, 12)	6 (3, 11)
**Prevalent disease, *n* (%)**			
** Diabetes**	364 (21)	179 (20)	185 (22)
** Hypertension**	1024 (58)	549 (60)	475 (55)
** Cardiovascular disease**	111 (6)	43 (5)	68 (8)
** Congestive heart failure**	35 (2)	16 (1.7)	19 (2.2)
**AHI, events per hour, median (IQR)**	21 (12, 35)	17 (11, 29)	26 (15, 44)
**AHI > 15, *n* (%)**	1175 (66)	523 (57)	652 (76)
**Average sleep time (min), mean (SD)**	392 (85)	406 (78)	378 (90)
**Stage N1 sleep (%), mean (SD)**	14.9 (9.3)	12.4 (7.3)	17.6 (10.5)
**Stage N3 sleep (%), mean (SD)**	9.8 (8.9)	12.6 (9.4)	7.1 (7.4)
**Stage REM sleep (%), mean (SD)**	17.9 (6.6)	18.8 (6.6)	17.0 (6.5)
**ESS>10, *n* (%)**	245 (14)	122 (13)	123 (14)
**Insomnia symptoms,^b^ *n* (%)**	616 (35)	349 (38)	267 (31)
**Short sleep duration (<6 h); *n* (%)**	537 (30)	223 (24)	314 (36)
**Sleep apnea severity metrics**			
** Hypoxic burden, %min h^−1^, median (IQR)**	42 (24, 77)	32 (21, 58)	54 (31, 94)
**Heart rate response (Δ HR), BPM *n* (%)**			
** Low Δ HR (<5.8)**	409 (23)	247 (27)	162 (19)
** High Δ HR (≥10.1)**	446 (25)	177 (19)	269 (32)
**Arousal intensity, mean (SD)**	4.8 (0.6)	4.9 (0.6)	4.7 (0.6)
**Event duration, sec, mean (SD)**	20.9 (4.1)	19.6 (4.1)	22.1 (5.3)
**OSA endotypes**			
** Collapsibility, %eupnea, median (IQR)**	20 (13, 28)	18 (11, 24)	24 (16, 34)
** Compensation, %eupnea, median (IQR)**	5.35 (3.28, 9.98)	5.26 (3.55, 9.13)	5.56 (2.70, 11.07)
**Loop gain (LG), mean (SD)**	0.58 (0.17)	0.57 (0.18)	0.59 (0.17)
**Arousal threshold, %eupnea, median (IQR)**	112 (105, 126)	109 (104, 119)	116 (108, 137)

Abbreviations: AHI, apnea hypopnea index; BPM, beats per minute; CES-D, Center for Epidemiological Studies Depression Scale; ESS, Epworth Sleepiness Scale; IQR, interquartile range.

a Depression symptoms assessed by the Center for Epidemiological Studies Depression Scale (CES-D).

b Insomnia symptom assessed by the Women’s Health Insomnia Rating Scale score ≥ 9.

Table 2 highlights the differences between participants with and without EDS. There were no differences in age, sex, smoking status, or CVD. Participants with EDS had modestly higher AHI and BMI values, and a higher prevalence of insomnia symptoms, short sleep duration, and diabetes. EDS also was associated with higher HB, shorter event duration, elevated loop gain, and higher arousal threshold. No differences by EDS were observed for airway collapsibility, neuromuscular compensation, or arousal intensity.

**Table 2:** Characteristics of the sleep study participants, by excessive daytime sleepiness (Epworth Sleepiness Scale [ESS] >10).

	ESS ≤ 10 *n* = 1538	ESS > 10 *n* = 245	*P*
**Age, yr, mean (SD)**	69.0 (9.1)	66.9 (8.9)	.61^a^
**Male, *n* (%)**	739 (48)	123 (50)	.53^b^
**Race/ethnicity, *n* (%)**			.001^b^
** White**	580 (37)	75 (31)	
** Chinese**	186 (12)	26 (11)	
** Black**	393 (26)	90 (37)	
** Hispanic**	379 (25)	54 (22)	
**BMI, kg m^−2^, mean (SD)**	28.7 (5.4)	30.3 (6.0)	.01^a^
**Smoking status, *n* (%)**			.43^b^
** Current**	109 (7)	21 (9)	
** Noncurrent**	1417 (93)	224 (91)	
**Depression symptoms, median (IQR)**	6 (2, 11)	8 (3,14)	.01^c^
**Prevalent disease, *n* (%)**			
** Diabetes**	299 (20)	65 (27)	.01^b^
** Hypertension**	876 (57)	148 (60)	.32^b^
** Any CVD**	100 (7)	11 (5)	.22^b^
**Short sleep duration (<6 h), *n* (%)**	440 (29)	97 (40)	.001^b^
**AHI, events/hr, median (IQR)**	20 (12, 34)	23 (14, 41)	.001^c^
**Insomnia symptoms,^d^ *n* (%)**	508 (33)	108 (44)	.001^b^
***Sleep apnea severity metrics***			
** Hypoxic burden, %min per hour, median, (IQR)**	42 (24,76)	44 (26, 88)	.01^c^
**Heart rate response (ΔHR), BPM, *n* (%)**			.08^c^
** Low Δ HR (<5.8)**	358 (24)	51 (21)	
** High Δ HR (≥10.1)**	371 (24)	75 (31)	
**Arousal intensity, mean (SD)**	4.8 (0.6)	4.8 (0.6)	.14^a^
**Event duration, sec, mean (SD)**	21.0 (5.0)	20.0 (4.0)	.001^a^
**OSA endotypic traits**			
**Collapsibility, % eupnea, median (IQR)**	20 (13, 28)	20 (12,.30)	.75^c^
**Compensation, % eupnea, median (IQR)**	5.30 (3.30, 9.73)	5.95 (3.25, 12.34)	.15^c^
**Loop gain (LG), mean (SD), median (IQR)**	0.57 (0.17)	0.62 (0.19)	.01^a^
**Arousal threshold, % eupnea**	111 (105, 125)	114 (106, 130)	.01^c^

Abbreviations: AHI, apnea hypopnea index; BPM, beats per minute; CES-D, Center for Epidemiological Studies Depression Scale; IQR, interquartile range.

a *t*-test (means [SD]).

b Chi-square test (%).

c Wilcoxon test (medians and interquartile ranges).

d Women’s Health Insomnia Rating Scale score of ≥9.

The variation in demographic and clinical characteristics associated with EDS was generally similar between males and females, with a few exceptions (Table S1). Both males and females with EDS had a higher prevalence of short sleep duration and insomnia. Among males, diabetes was more prevalent in those with EDS.

### Associations of individual traits and sleepiness

#### Disease severity metrics

In the overall sample, an interquartile increase in HB (from 24.3 to 77.1%min/h) was associated with a 28% higher prevalence ratio of EDS (PR: 1.28, 95% CI: 1.10, 1.51) after adjusting for age, sex, race and ethnicity, and smoking, corresponding to an absolute difference of 3.66 percentage points higher predicted prevalence (95% CI 1.43, 6.18) (Table S2). This association did not materially change after additionally adjusting for short sleep duration but weakened after accounting for comorbidities (Table 3). Compared to midrange levels, elevated ΔHR was associated with a 32% higher prevalence ratio of EDS (PR = 1.32, 95% CI 1.01, 1.73), corresponding to an absolute difference of 3.62 percentage points in predicted prevalence (95% CI −0.57, 8.46), and only slightly attenuated with further adjustments. No associations were found between event duration or arousal intensity with EDS in the overall sample.

**Table 3:** Prevalence ratios (PRs)^a^ and 95% confidence interval (CI) for the associations between OSA endo-phenotypes with excessive daytime sleepiness (Epworth Sleepiness Scale > 10), sex-combined sample.

	Model 1	Model 2	Model 3
**Physiological severity metrics**	**PR (95% CI)**	**PR (95% CI)**	**PR (95% CI)**
** Hypoxic burden (HB), IQR^b^**	**1.28 (1.10, 1.51)**	**1.26 (1.08, 1.48)**	1.17 (1.00, 1.38)
**Heart rate (HR) response^c^**			
** Δ HR (low vs. midrange)**	1.07 (0.79, 1.46)	1.08 (0.79, 1.47)	1.10 (0.81, 1.50)
** Δ HR (high vs. midrange)**	**1.32 (1.01, 1.73)**	1.31 (1.00, 1.71)	1.29 (0.99, 1.68)
** Arousal intensity, IQR**	0.94 (0.81, 1.09)	0.94 (0.81, 1.09)	0.96 (0.83, 1.11)
** Event duration, IQR**	0.88 (0.76, 1.01)	0.89 (0.76, 1.01)	0.91 (0.79, 1.04)
**OSA endotypic traits**			
** Collapsibility, IQR**	1.07 (0.96, 1.20)	1.06 (0.95, 1.19)	1.05 (0.94, 1.17)
** Loop gain, IQR**	**1.26 (1.12, 1.42)**	**1.25 (1.11, 1.41)**	**1.19 (1.05, 1.35)**
** Arousal threshold, IQR^d^**	1.14 (0.98, 1.33)	1.13 (0.97, 1.32)	1.12 (0.97, 1.30)

Model 1: (base model) includes age (years), sex, race and ethnicity (ref. White), and current smoking. Model 2: Model 1 plus short sleep duration (<6 h vs. ≥6 h). Model 3: Model 2 plus BMI (kg m^−2)^, CES-D score (continuous and standardized), diabetes, and prevalent CVD. Bolded values highlight 95% confidence intervals that do not overlap 1.0.

a IQR (interquartile range): Interquartile prevalence ratios (PRs) are shown across the interquartile ranges. For example, a PR of 1.28 for hypoxic burden represents the ratio of predicted prevalence of EDS comparing the 75th versus the 25th percentile of hypoxic burden (77.1 vs. 24.3%min h^−1^), holding all other covariates constant. A HB of 24%min h^−1^ represents 6 min of 4% desaturation per hour of sleep or 3 min of 8% desaturation per hour of sleep.

b Hypoxic burden modeled after logarithmic transformation.

c Heart rate (HR) response was categorized as low (<5.8 BPM, approximately the 25th percentile) and high (>10.1 BPM, roughly the 75th percentile), with the midrange as the reference group.

d Arousal threshold modeled after square root-transformation.

#### Endotypes

An interquartile increase in LG was associated with a 1.26-fold higher relative prevalence of EDS (PR: 1.26, 95% CI: 1.12, 1.42), with little change with further adjustment (Table 3), corresponding to an absolute difference of 2.65 percentage points (95% CI: 0.81, 4.46). Arousal threshold showed a 14% higher relative prevalence of EDS, which was imprecise (95% CI: 0.98, 1.33).

**Figure 1:**
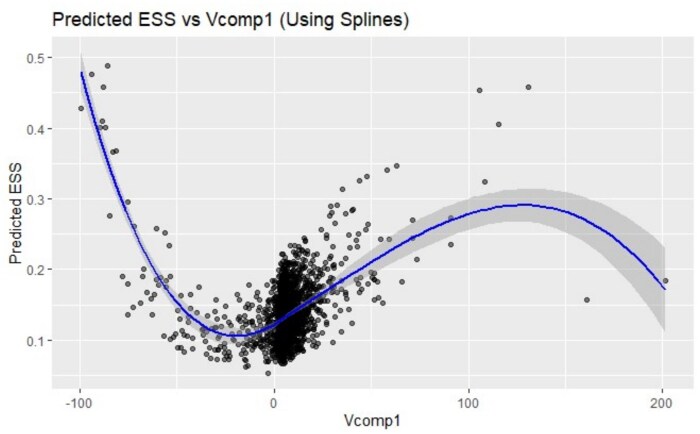
Smooth spline curve showing the association between compensation and excessive daytime sleepiness (Epworth Sleepiness Score, ESS). Predicted prevalence of excessive daytime sleepiness (ESS > 10) across compensation values from a Poisson regression model with cubic splines (5 knots) and robust standard errors. The model is adjusted for age, sex, race and ethnicity, and smoking. The solid line indicates the fitted risk; the shaded band represents the 95% CI. The Y-axis displays the Predicted Probability of Sleepiness (ESS > 10). The individual data points represent model-predicted probability values based on compensation plus other covariates. The fitted curve shows a U-shaped relationship, with the lowest predicted ESS probabilities near the midrange of compensation and higher probabilities at both low and high extremes.

In Figure 1, we present the adjusted relationship between compensation and predicted EDS using cubic splines within a Poisson regression model. The resulting curve displays a U-shaped pattern, showing higher predicted EDS values at both the lower and higher values of compensation. However, in the overall sample (both sexes), the CIs for the PR for estimates for the interquartile values overlap one (Table 4). In contrast, in sex-stratified analyses, increased compensation was associated with a 78% (95% CI: 1.18. 2.68) increased prevalence ratio for EDS in females, with no evidence of an association in males; *P* interaction (for sex difference) = .08).

**Table 4:** Prevalence ratios (PRs) and 95% CI of the association between compensation and excessive daytime sleepiness (Epworth Sleepiness Scale > 10), for the whole sample and by sex.

	Model 1	Model 2	Model 3
**Compensation** ^a^	**PR (95% CI)**	**PR (95% CI)**	**PR (95% CI)**
**All**			
**Low tertile**	1.15 (0.85, 1.56)	1.14 (0.84, 1.54)	1.16 (0.86, 1.56)
**High tertile**	1.32 (0.98, 1.76)	1.30 (0.97, 1.74)	1.30 (0.97, 1.74)
**Female**			
**Low tertile**	1.33 (0.87, 2.03)	1.31 (0.85, 2.00)	1.32 (0.86, 2.02)
**High tertile**	**1.78 (1.18, 2.68)**	**1.74 (1.16, 2.62)**	**1.70 (1.14, 2.53)**
**Male**			
**Low tertile**	0.94 (0.62, 1.42)	0.93 (0.61, 1.41)	0.95 (0.62, 1.44)
**High tertile**	0.94 (0.62, 1.42)	0.93 (0.62, 1.41)	0.97 (0.64, 1.48)

a Reference: the middle tertile of compensation.

Model 1: (base model) includes age (years), sex, race and ethnicity (ref. White), and current smoking. Model 2: Model 1 plus short sleep duration (<6 h vs. ≥6 h). Model 3: Model 2 plus BMI (kg m^−2^), depression symptoms, diabetes, and prevalent CVD. Bolded values indicate estimates with 95% confidence intervals that do not overlap 1.0.

### Sex-specific associations

Table 5 shows the sex-specific results for associations with EDS for endo-phenotypes other than compensation. In females, an association between event duration and EDS emerged, showing a 26% lower relative prevalence of EDS with an increase in event duration from the 25th to the 75th percentile (PR: 0.74, 95% CI: 0.60, 0.92); *P* interaction (for sex difference) = .03. Associations for HB and ΔHR tended to be stronger for males (with male-specific CIs greater than one), although tests for sex interaction were not significant. No differences were noted after adjusting for sleep duration (data not shown); however, adjustments for comorbidities slightly diminished most sex-specific associations (Table S3).

**Table 5:** Prevalence ratios (PRs)^a^ and 95% confidence interval (CI) for the associations between OSA endo-phenotypes with EDS, by sex.

	Female	Male	*P* (sex interaction)
**Physiological responses**	**PR (95% CI)**	**PR (95% CI)**	
** Hypoxic burden (HB), IQR**	1.12 (0.92, 1.36)	**1.37 (1.10, 1.70)**	.19
**Heart rate (HR) response^b^**			
** Δ HR (low vs. midrange)**	1.02 (0.69, 1.53)	1.13 (0.69, 1.83)	.97
** Δ HR (high vs. midrange)**	1.12 (0.73, 1.70)	**1.46 (1.02, 2.09)**	.37
** Arousal intensity**	0.90 (0.74, 1.10)	1.00 (0.81, 1.22)	.86
** Event duration**	**0.74 (0.60, 0.92)**	0.98 (0.82, 1.17)	.03
**OSA endotypic traits**			
** Collapsibility, IQR**	0.94 (0.77, 1.15)	1.12 (0.96, 1.30)	.10
** Loop gain (LG), IQR^c^**	**1.21 (1.03, 1.43)**	**1.28 (1.07, 1.53)**	.39
**Arousal threshold, IQR^d^**	1.11 (0.92, 1.35)	1.14 (0.92, 1.42)	.58

Models adjusted for age (years), race-ethnicity (ref. White), and current smoking. Bolded values indicate 95% confidence intervals that do not overlap 1.0.

a IQR (interquartile range): interquartile prevalence ratios (PRs) across the interquartile ranges.

b Heart rate (HR) response was categorized as low (<5.8 BPM, approximately the 25th percentile) and high (>10.1 BPM, roughly the 75th percentile), with the midrange as the reference group.

c Hypoxic burden was modeled as logarithmically transformed.

d Arousal threshold modeled after square root-transformation.

Additional adjustments for psychotropic medications and depressive symptoms or alcohol use changed the effect estimates minimally (by 0 to 2%; data not shown).

## Discussion

This study examined the associations of sleepiness with OSA endo-phenotypes in a large, diverse, community-based sample of individuals. In the overall sample, high hypoxic burden, high heart rate response to respiratory events, and elevated loop gain were each associated with a roughly 25%-30% increased prevalence ratio of excessive daytime sleepiness after adjusting for demographic factors, smoking, and short sleep duration. Sex-specific analyses suggested that the associations of hypoxic burden and heart rate response with sleepiness were stronger in males. In female-specific analyses only, sleepiness was associated with shorter event duration and elevated compensation. These findings support the importance of considering endo-phenotypic differences when interpreting the heterogeneity and symptom profile of OSA. The observed sex differences suggest that sleepiness may be a marker for different OSA-related patho-etiological pathways in males and females.

Only limited research has examined the association between mechanistic traits (endotypes) and EDS. In a clinic-based study, Cheng  et al. identified a sleepiness symptom cluster that was associated with an endotype cluster characterized by high loop gain and increased collapsibility.^28^ In contrast to these findings, we did not detect an association between collapsibility and EDS, possibly due to differences in analytic methods and population characteristics. We did, however, confirm an association between loop gain and EDS, which was observed in both males and females. High loop gain, indicative of a sensitive ventilatory control system, is more common in older individuals and those with obesity or heart failure.^14^ In our sample, only 2% had known heart failure, suggesting that high loop gain may be informative for understanding OSA subtype differences across general samples of individuals with OSA.

We also showed that EDS was associated with two measures of physiological severity-hypoxic burden and elevated heart rate response to respiratory events. Both markers are associated with adverse outcomes and predict continuous positive airway pressure (CPAP) response. A high hypoxic burden, which quantifies the sleep-apnea-specific depth and duration of overnight hypoxemia, predicted mortality and CVD in several cohorts.^13^^,^^29^ Analyses from the Sleep Heart Health Study (SHHS) also reported that hypoxic burden was higher among individuals with moderate-severe EDS than those without EDS.^30^ Our analyses confirm an association between hypoxic burden and sleepiness and suggest that this association is stronger in males. A high (vs. an intermediate) heart rate response to respiratory events, reflecting cardiac autonomic reactivity, also was shown to predict CVD and mortality in large cohort studies^15^ and to predict blood pressure improvement with CPAP.^31^ A recent secondary analyses of several trials of CPAP intervention also reported that both hypoxic burden and elevated heart rate response appeared to be “high risk markers” that identify OSA subgroups likely to experience reduced incidence of major cardiovascular events with CPAP treatment.^32^

There is growing evidence for sex differences in OSA-related outcomes as well as polysomnographic features of OSA, with females having shorter events with less oxygen desaturation, more hypopneas and fewer apneas, and a lower arousal threshold.^7^ The current study showed that hypoxic burden was higher and elevated ΔHR was more common in males; these metrics also tended to be more strongly associated with EDS in males compared to females. We also identified an association between shorter event duration and EDS only in females. In the SHHS, individuals with shorter event duration were more frequently younger, female, Black, and current smokers,^16^ which was similar to the findings in the current study. A short event duration phenotype may reflect a propensity to sleep fragmentation and elevated sympathetic activity, and is predictive of increased mortality.^16^ Among females only, we also observed a higher prevalence ratio of EDS in association increased neuromuscular compensation. It is possible that females may recruit upper airway muscles more intensely, which can fragment sleep and contribute to daytime sleepiness. These findings suggest that EDS may be associated with different OSA-related physiological features in males and females—specifically, EDS appears to associate with hypoxia-related mechanisms in males and with traits that may result in sleep fragmentation in females.

A surprising finding was that neither arousal threshold or arousal intensity was associated with EDS. These findings may reflect the complexity of EEG-based metrics that may change over time because of an untreated disease. For example, in the SHHS, individuals with a higher number of events ending in arousal reported less sleepiness, and the arousal index declined over time in individuals with severe untreated OSA.^33^

The bases for the observed associations between EDS and endo-phenotypes are not known. However, endophenotypic features of OSA such as those related to hypoxia and arousal response may influence sympathetic activation and levels of inflammatory cytokines. Evidence from animal models and human studies implicates inflammatory cytokines in sleep regulation, sleepiness, and metabolic dysfunction.^34^ Inflammation has been shown to be bidirectionally associated with OSA—and specifically has been associated with the OSA sleepy subtype.^35^ A causal role of inflammation in increasing risk for the sleepiness within individuals with OSA is supported by a Mendelian Randomization analysis that showed that an elevation in a genetic marker of the inflammatory protein, C-reactive protein, predicted a 40% higher risk of OSA with EDS, but was not associated with OSA without EDS.^36^ Research is needed to study the hypothesis that EDS results from inflammatory and autonomic nervous system responses secondary to, or associated with, OSA endo-phenotypes.

As HB, ΔHR, LG, and short event duration are markers of increased morbidity and decreased survival,^13^^,^^15^^,^^16^ our analyses suggest that these OSA endo-phenotypic markers may partly explain prior associations reported between sleepiness and CVD. A large clinic-based sample reported that HB but not symptom characteristics predicted incidence of major adverse cardiac events (MACE) in mutually adjusted analyses.^37^ In contrast, a secondary analysis of the SHHS found that the excessively sleepy symptom subtype was a risk factor for incident MACE, even after considering HB (although HB and not symptoms was associated with CVD mortality in mutually adjusted models).^30^ These results, including our findings that no one endo-phenotype explained a large portion of the variance in EDS, underscore the complexity in the pathophysiology of OSA and the need for future research that combines symptom-cluster phenotyping with more detailed physiological signals to better understand these relationships.

Our results should be interpreted in light of the known complexity of EDS. Apart from OSA, EDS is predictive of vascular dementia,^38^ CVD, stroke, and mortality.^39^ EDS is also associated with a range of other exposures, such as insufficient sleep,^40^ medication or substance use,^41^ mood disorders,^42^ central nervous system disorders of hypersomnolence, and metabolic or inflammatory processes that affect the brain’s sleep–wake centers.^43^ In our analyses, short sleep, medications, and mood disorders did not substantively confound associations with EDS. EDS also displays genetic heterogeneity.^44^ Future use of genetic instruments and multi-modal markers may further elucidate clinically important EDS subtypes and identify which ones are specific to OSA-related pathophysiology.

Variations in how EDS is assessed and perceived, population differences due to ascertainment (e.g., random sampling, symptom-driven, or co-morbidity–driven), and the socio-demographic and health characteristics of the samples may influence the specificity of EDS as a symptom of OSA and its utility as a disease subtype. While the ESS is the most common tool for quantifying EDS in sleep clinics and has high internal consistency (alpha Cronbach: 0.82),^45^ our 10 year data from 115 participants in the MESA study show only moderate stability (intraclass correlation coefficient 0.57; unpublished) and may be less valid for older individuals and females.^46^ Therefore, our observed associations between EDS and endo-phenotypes may be interpreted as conservative lower-bound estimates. In addition, we cannot exclude the possibility that sex differences in responses to the ESS questionnaire influenced our findings.

The strength of this study lies in the analysis of a large, diverse, community-based sample with a wide range of OSA severity. Measurements included standardized polysomnography, the use of validated advanced polysomnography signal analyses to generate OSA endotypes and severity measures, and information on a wide range of covariates collected either at the time of or before participants underwent polysomnography following research protocols. Limitations include the reliance on single-night polysomnography (and potential misclassification due to night-to-night variation), the use of non-invasive estimates of endotypes rather than gold-standard invasive measurements, and assessment of EDS with a single tool and summary score (the ESS) that may vary in sensitivity by sex. Similarly, there is a need to further validate endotype assessments in females. Our analyses of sex-specific differences were limited by the limited statistical power to detect interaction and should be interpreted as hypothesis generating. Our findings also may not be generalizable to younger or clinic-based samples (including those with more severe symptom profiles and co-morbidity patterns).

## Conclusion

In summary, we identified novel associations between sleepiness and several OSA endo-phenotypes—both physiological severity and endotypic metrics—in a large, community-based cohort; our analyses also suggested variations in associations by sex. The results provide insights into physiological risk factors underlying variation in sleepiness among individuals with OSA and suggest that polysomnographic measures of OSA mechanisms and severity—potentially combined with symptom reports—may be useful for identifying high-risk subgroups and uncover mechanisms explaining the associations of symptoms with adverse health outcomes.

## Supplementary Material

aaoag078_Supplementary_Data

## Data Availability

This article has an online supplement, which is accessible at the Supplements Tab.
