# Supplementary material for: Associations of excessive sleepiness with sleep apnea physiological endo-phenotypes: the multi-ethnic study of atherosclerosis
Source: Ann Am Thorac Soc. 2026 Apr 7;23(7):1067–76. doi: 10.1093/annalsats/aaoag078 (PMC13315775; doi:10.1093/annalsats/aaoag078)
Supplement: aaoag078_Supplementary_Data [file aaoag078_supplementary_data.zip › Supplemental materials FINAL NO TRACK CHANGES.docx]

**On-Line Supplement**

**Associations of Excessive Sleepiness with Sleep Apnea Physiological Endo-phenotypes: The Multi-Ethnic Study of Atherosclerosis**

Cecilia Castro-Diehl, M.D., Dr.P.H.^1^; Raichel Alex, Ph.D.^1^; Ali Azarbarzin, Ph.D.^1^; Ying Zhang, Ph.D.^1^; Andrew Wellman, M.D., Ph.D.^1^; Tianyi Huang, D.Sc.^2^; Scott Sands, Ph.D.^1^; Susan Redline, M.D., M.P.H.^1^

^1^Division of Sleep and Circadian Disorders, Departments of Medicine and Neurology, Brigham and Women’s Hospital, Harvard Medical School; Brigham and Women's Hospital, Boston, Massachusetts, USA

^2^Laboratory of Epidemiology and Population Sciences, Intramural Research Program, National Institute on Aging, Baltimore, Maryland, USA

**Supplemental Tables**

**Table S1**. Characteristics of Females and Males of the MESA Sleep Study, by sex and Excessive Daytime Sleepiness (Epworth Sleepiness Scale >10)

|  | Female  (n = 921) | | Male  (n = 862) | |
| --- | --- | --- | --- | --- |
|  | **ESS ≤ 10**  **(n=799)** | **ESS >10**  **(n=122)** | **ESS ≤10**  **(n=739)** | **ESS > 10**  **(n=123)** |
| Age, yr, n (%) | 69.0 (9.0) | 67.1 (9.5) | 68.9 (9.3) | 67.1 (8.6) |
| Race/Ethnicity, n (%) |  |  |  |  |
| White | 294 (37%) | 41 (33%) | 286 (39%) | 34 (28%) |
| Chinese | 92 (12%) | 7 (6%) | 94 (13%) | 19 (15%) |
| Black | 215 (27%) | 50 (41%) | 178 (24%) | 40 (33%) |
| Hispanic | 198 (25%) | 24 (20%) | 181 (24%) | 30 (24%) |
| BMI, kg/m^2^ | 29.3 (6.1) | 30.9 (6.8) | 28.1 (4.3) | 29.7 (5.0) |
| Smoking status, n (%) |  |  |  |  |
| Current | 49 (6%) | 15 (12%) | 60 (8%) | 6 (5%) |
| Noncurrent | 743 (94%) | 107 (88%) | 674 (92%) | 117 (95%) |
| Depression Symptoms^(1)^, median (IQR) | 6 (3,12) | 8 (3,15) | 6 (2,10) | 6.5 (3,13) |
| Prevalent disease, n (%) |  |  |  |  |
| Diabetes | 154 (20%) | 25 (21%) | 145 (20%) | 40 (33%) |
| Hypertension | 472 (59%) | 77 (63%) | 404 (55%) | 71 (58%) |
| Any CVD | 40 (5%) | 3 (2%) | 60 (8%) | 8 (7%) |
| Short Sleep Duration, < 6h, n, % | 181 (23%) | 42 (34%) | 259 (35%) | 55 (45%) |
| AHI^(2)^, events/hr, median (IQR) | 16.8 (10.4,28.4) | 17.7 (11.5, 32.5) | 24.8 (14.8, 42.9) | 33.2 (16.9, 52.9) |
| Insomnia Symptoms^(3)^, n (%) | 296 (37%) | 53 (43%) | 212 (29%) | 55 (45%) |

IQR: Interquartile range; ^(1)^ CES-D, Center for Epidemiological Studies Depression Scale ; ^(2)^ AHI: Apnea hypopnea index; ^(3)^  Women’s Health Insomnia Rating Scale score of ≥9 [^47^](#_ENREF_47)

**Table S2.** **Adjusted* Predicted Prevalences and Prevalence Differences with 95% Confidence Intervals (CI) for associations between OSA endo-phenotypes and Excessive Daytime Sleepiness (ESS > 10)**

| **Exposure** | **Contrast** | **Prevalence (reference (**p_0_**)**  **(%)** | **Comparator**  **Prevalence (**p_1_**) (%)** | **Risk Difference (95% C.I)** |
| --- | --- | --- | --- | --- |
| **Hypoxic Burden** | |  |  |  |
|  | Q75 vs Q25 | 12.01 | 15.6 | 3.66 (1.43, 6.18) |
| **∆HR** |  |  |  |  |
|  | High vs Middle | 12.6 | 16.2 | 3.62 (-0.57, 8.46) |
|  | Low vs Middle | 12.6 | 13.8 | 1.20 (-3.11, 5.57) |
| **Arousal Intensity** | |  |  |  |
|  | Q75 vs Q25 | 14.2 | 13.4 | -0.84 (-3.29, 1.37) |
| **Event Duration** | |  |  |  |
|  | Q75 vs Q25 | 14.6 | 13.2 | -1.34 (-3.82, 1.26) |
| **Collapsibility** | |  |  |  |
|  | Q75 vs Q25 | 13.2 | 14.3 | 1.14 (-0.62, 2.66) |
| **Loop Gain** | |  |  |  |
|  | Q75 vs Q25 | 12.2 | 14.9 | 2.65 (0.81, 4.46) |
| **Arousal Threshold** | |  |  |  |
|  | Q75 vs Q25 | 13.1 | 15.0 | 1.94 (-0.03, 4.15) |

*Models include age (years), sex, race-ethnicity (ref. White), and current smoking

p_0_ = predicted prevalence at the reference exposure (e.g. 25^th^ percentile or “Middle range”)

p_1_ = predicted prevalence at the higher or lower exposure level (e.g. 75^th^ percentile, “High”)

RD = Risk Difference: absolute differences in predicted prevalence

Risk difference = p_1_ – p_0_ , expressed in percentage points with 95% CIs from parametric bootstrap resampling.

Example: Participants with higher hypoxic burden (75^th^ percentile) had a predicted sleepiness prevalence about 3.7 percentage points higher (RD = 3.7 pp; 95% CI 1.4-6.2) than those with low hypoxic burden in the model adjusted for age, sex, race-ethnicity, and smoking.

**Table S3**. Prevalence Ratios (PRs)* and 95% Confidence Interval (CI]) for the Associations between OSA endo-phenotypes with EDS and by sex, adjusted for multiple co-morbidities^¥^

|  | **Female** | **Male** |
| --- | --- | --- |
| ***Physiological severity metrics*** | **PR (95%CI)** | **PR (95%CI)** |
| Hypoxic burden (HB) | 1.04 (0.86, 1.27) | 1.24 (0.99, 1.55) |
| Heart Rate (HR) response** |  |  |
| **Δ** HR (low vs midrange) | 1.08 (1.72, 1.62) | 1.08 (0.67, 1.75) |
| **Δ** HR (high vs midrange) | 1.13 (0.74, 1.72) | 1.41 (0.99, 2.00) |
| Arousal intensity, IQR | 0.94 (0.78, 1.15) | 0.99 (0.81, 1.20) |
| Event duration, IQR | **0.75 (0.61, 0.93)** | 1.01 (0.85, 1.20) |
| ***OSA endotypic traits*** |  |  |
| Collapsibility, IQR | 0.92 (0.75, 1.12) | 1.10 (0.95, 1.27) |
| Loop gain (LG), IQR ^§^ | 1.16 (0.97, 1.39) | **1.22 (1.03, 1.45)** |
| Arousal threshold, IQR^ɣ^ | 1.10 (0.91, 1.33) | 1.13 (0.92, 1.39) |

^¥^Models adjusted for age (years), race and ethnicity (ref. White), current smoking, short sleep duration (<6h vs. ≥6h), BMI (Kg/m^2^, standardized), CES-D score (continuous and standardized), diabetes, and prevalent CVD

* PR: Prevalence Ratios across the inter-quartile ranges (IQR).

**Heart rate response was categorized as low (<5.8 BPM, approximately the 25th percentile) and high (> 10.1 BPM, roughly the 75th percentile), with the midrange as the reference group

^§^Hypoxic burden was modeled as logarithmically transformed.

^ɣ^Arousal threshold modeled after square root-transformation.
